# Multidimensional analysis of gene expression reveals TGFB1I1-induced EMT contributes to malignant progression of astrocytomas

**DOI:** 10.18632/oncotarget.2518

**Published:** 2014-12-31

**Authors:** Yanwei Liu, Huimin Hu, Kuanyu Wang, Chuanbao Zhang, Yinyan Wang, Kun Yao, Pei Yang, Lei Han, Chunsheng Kang, Wei Zhang, Tao Jiang

**Affiliations:** ^1^ Department of Molecular Neuropathology, Beijing Neurosurgical Institute, Capital Medical University, Beijing, China; ^2^ Department of Neurosurgery, Beijing Tiantan Hospital, Capital Medical University, Beijing, China; ^3^ Laboratory of Neuro-Oncology, Tianjin Neurological Institute, Tianjin Medical University, Tianjin, China; ^4^ Department of Neurosurgery, The First Affiliated Hospital of Dalian Medical University, Dalian Medical University, Dalian, China; ^5^ Department of Molecular Neuropathology, Beijing Sanbo Brain Hospital, Capital Medical University, Beijing, China; ^6^ Chinese Glioma Cooperative Group (CGCG), China; ^7^ China National Clinical Research Center for Neurological Diseases, China; ^8^ Center of Brain Tumor, Beijing Institute for Brain Disorders, Beijing, China

**Keywords:** Astrocytomas, Malignant progression, Gene expression, TGFB1I1, EMT

## Abstract

Malignant progression of astrocytoma is a multistep process with the integration of genetic abnormalities including grade progression and subtypes transition. Established biomarkers of astrocytomas, like *IDH1* and *TP53* mutation, were not associated with malignant progression. To identify new biomarker(s) contributing to malignant progression, we collected 252 samples with whole genome mRNA expression profile [34 normal brain tissue (NBT), 136 grade II astrocytoma (AII) and 82 grade III astrocytoma (AIII)]. Bioinformatics analysis revealed that EMT-associated pathways were most significantly altered along with tumor grades progress with up-regulation of 17 genes. Up-regulation of these genes was further confirmed by RNA-sequencing in 128 samples. Survival analysis revealed that high expression of these genes indicates a poor survival outcome. We focused on *TGFB1I1* (TGF-β1 induced transcript 1) whose expression correlation with WHO grades was further validated by qPCR in 6 cell lines of different grades and 49 independent samples (36 AIIs and 13 AIIIs). High expression of TGFB1I1 was found associated with subtype transition and EMT pathways activation. The conclusion was confirmed using immunohistochemistry in tissue microarrays. Studies *in vitro* and *in vivo* using TGF-β1 and TGFB1I1 shRNA demonstrated that TGFB1I1 is required for TGF-β stimulated EMT that contributes to malignant progression of astrocytomas.

## INTRODUCTION

Astrocytomas are the most prevalent primary brain tumor and characterized by invasive and rapid growth. Tumor cells achieve rapid invasion and long-distance migration from the tumor mass into the normal brain tissue, and these processes are responsible for tumor recurrence. The invasion of tumor cells increases gradually with tumor grade progress. At present, histomorphology remains the only criterion for the diagnosis of astrocytomas. According to the world health organization (WHO) standards, the grading is based on the presence or absence of nuclear atypia, mitosis, vascular proliferation, and necrosis [1]. Low grade astrocytomas with characteristic of nuclear atypia or mitosis have longer survival but ultimately transform to a higher grade tumor with increasing malignancy (vascular proliferation or necrosis). However, there also exists different malignant degree on the equal grade that corresponds to different prognosis [2, 3]. Therefore, the malignant phenotype of astrocytomas cannot be well characterized by the current grading system.

Advances in molecular genetics are challenging the traditional morphological categorization of tumors. The theory that glioma is a result of polygenic disorder is increasingly being recognized. Gene expression abnormalities are associated with progression of gliomas and can differentiate not only among histologic subtypes but also between low and high grade gliomas [4, 5]. Molecular alterations on primary GBM (arising de novo) have been studied thoroughly. But II-III grade astrocytomas and secondary GBM (arising from lower-grade gliomas) are less well-characterized in tumor formation and progression. Major findings in low grade astrocytoma include frequent activating mutations in *IDH1* and *TP53*, and *RB1* inactivation [6–8]. Recently, Jiao et al. found that ATRX inactivation is linked to mutations in *TP53* and *IDH1* in low grade gliomas [9]. These classical alterations were generally considered as the earliest genetic abnormalities in the development of astrocytomas. But the high frequency of these alterations are already present in low grade gliomas (AII) and the frequency does not increase (even decrease) in high grade gliomas (AIII or GBM) suggesting that they might not associated with malignant progression of astrocytomas. More importantly, there also exists another malignant progression on the equal grade of tumors (subtypes transition). Different subtypes have different malignant phenotypes that were also resulted from many genetic alterations [3, 10]. Therefore, discovery of new driver markers would help to understand molecular mechanisms of astrocytomas progression.

The aim of the present study was to identify genetic alterations involved in the malignant progression of astrocytomas. Secondary GBM not to be included in the study due to patients undergo a second operation or chemoradiotherapy that might affect gene expression [11]. The established biomarkers of astrocytomas, like *IDH1* and *TP53* mutation, were not associated with malignant progression though could predict survival in the present or previous studies [12]. To identify new biomarker(s), we collected and analyzed 252 samples with whole genome expression profile (34 NBTs, 136 AIIs and 82 AIIIs). The candidate genes which were up-regulated with increasing tumor grades were further confirmed on 128 samples with RNA-sequencing (57 AIIs and 71 AIIIs). Finally, we focused on *TGFB1I1* which was a TGF-β1 induced transcription factor involved in the EMT process. In addition, TGFB1I1 might be associated with subtype transition and could be used as serviceable marker for mensenchymal astrocytoma. The transcriptional and the protein level of TGFB1I1 were further validated on additional samples by qPCR and IHC. Finally, studies in vivo and vitro demonstrated that TGF-β1-inducible TGFB1I1 is required for regulation of cell migration and invasion and is an important regulator of TGF-β stimulated EMT. This finding is new opportunity for understanding the fundamental basis for malignant progression of astrocytomas and also provide novel interfering target for shutting down astrocytomas progression.

## RESULTS

### The established biomarkers were not associated with grade progression

At present, many reliable molecular markers, such as *TP53* and *IDH1* mutation, have been accepted as early alterations in astrocytomas development [6, 7]. In this study, we asked whether these master markers are changed with increasing tumor grades. By application of various detection techniques, we counted *PTEN* mutation, *IDH1* R132 mutation, 1p19q loss, *TP53* mutation, *MGMT* promoter methylation and *EGFR* amplification in CGGA database (Table 1). We found that *PTEN* mutation and *MGMT* promoter methylation are significantly increased with increasing tumor grade from 3.5% and 30.4% in AII to 17.6% and 62.5% in AIII. 1p19q loss and EGFR amplification which were mainly identified in oligodendrogliomas and primary GBM were not significantly different in different grades of astrocytoma. However, the highest alterations, *IDH1* and *TP53* mutation, were significantly decreased from 80% and 50% in AII to 39.4% and 27.8% in AIII. These data demonstrated that *PTEN* mutation might play driver role in grade progression of astrocytomas, but this alteration was only observed in a small minority of patients (4%). The data that high frequency of *IDH1* and *TP53* mutation are already present in AII and the frequency does not increase (even decrease) in AIII suggests that they might be not associated with malignant progression of astrocytomas, especially in progression from AII to AIII.

**Table 1:** Established biomarkers in different grade astrocytomas

Variables	NBT	AII	AIII	*P* value
***PTEN* mutation**	NA	5/140 (3.5%)	3/17 (17.6%)	<0.05
***IDH1* mutation**	NA	131/182 (80%)	13/33 (39.4%)	<0.05
***1p19q* loss**	0/6	12/217 (5.5%)	6/40 (15%)	>0.05
***TP53* mutation**	NA	67/149 (50.0%)	5/18 (27.8%)	<0.05
***MGMT* methylation**	NA	7/23 (30.4%)	15/24 (62.5%)	<0.05
***EGFR* amplification**	0/6	5/217 (2.3%)	2/40 (5%)	>0.05

### Significant changes in gene expression during grade progression of astrocytoma

**Figure 1:**
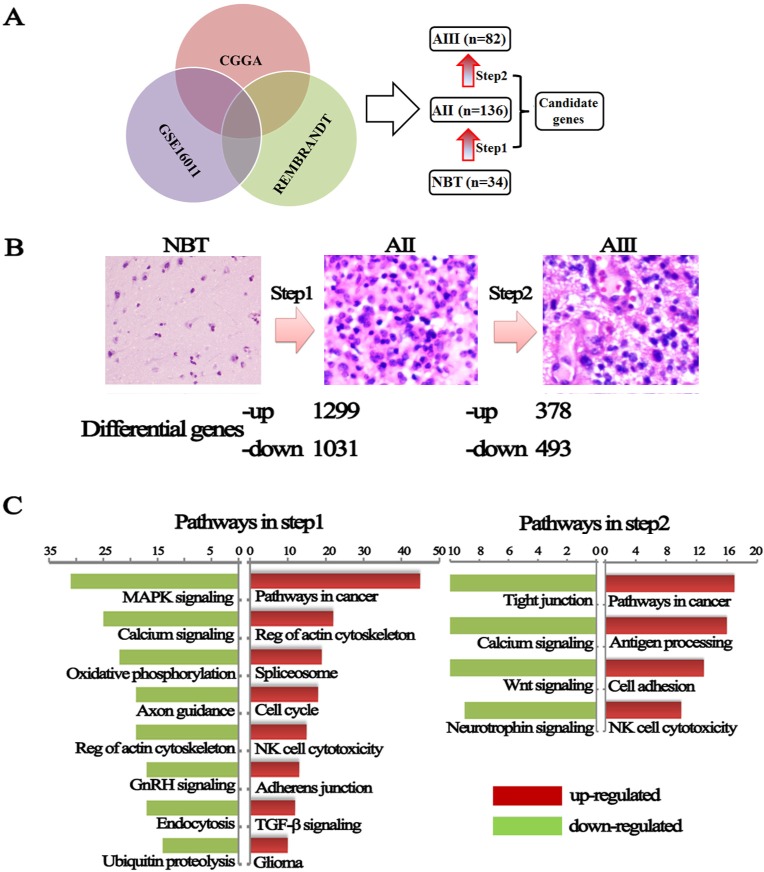
Changes in genes expression from NBT to grade II and grade II to grade III astrocytomas **(A)** Differential genes were obtained from overlapping CGGA, GSE16011 and REMBRANDT databases. **(B)** A hematoxylin and eosin stained section was performed to determine the pathological type and grade and dysregulated genes were divided into up-regulated and down-regulated during malignant progression of astrocytomas. **(C)** KEEG pathways analysis performed using these differential expression genes on the DAVID website.

To identify new gene(s) contributing grade progression, we compared genome expression between NBT, AII and AIII. 252 samples with gene expression profile were obtained from our institution (CGGA: 5 NBTs, 58 IIAs and 8 IIIAs) and 2 external independent databases of glioma (GSE16011: 8 NBTs, 13 IIAs and 16 IIIAs; REMBRANDT: 21 NBTs, 65 AIIs and 58 AIIIs) (Figure-1A). A total of 29421 probes (19416 genes), 40547 probes (19596 genes) and 40547 probes (19596 genes) were available in CGGA, GSE16011 and REMBRANDT databases, respectively. To avoid discrepancies due to different platforms, the data were analyzed separately within each database. After overlapping the analyzed data, a total of 1299 up-regulated genes and 1031 down-regulated genes were identified in AII compared with NBT (step1). 378 up-regulated and 493 down-regulated genes were identified in the step2 from AII to AIII (Figure-1B, supplementary table 2). To avoid any confusion in the data induced by one gene with more than one probe, the probes were consistent from NBT to AII, to AIII.

To further understand the changes in differentially genes and pathways of astrocytomas, Gene-set enrichment analysis were performed using a comprehensive set of functional annotation tool (The Database for Annotation, Visualization and Integrated Discovery, DAVID) [13]. As illustrated in Figure-1C, up-regulated gene expression profiles in AII, in comparison to NBT, were more strongly enriched in pathways related to cancer (sustained angiogenesis, VEGF signaling, evading apoptosis, and proliferation) and regulation of actin cytoskeleton, TGF-β-signaling pathway and glioma pathway. Similarly, pathways in cancer and cell adhesion molecules (CAMs) are also the main up-regulated pathways in AIII as compared to AII. The up-regulated pathways (actin cytoskeleton, adhesion molecules and TGF-β) suggested that grade progression of astrocytomas might result from obtaining the abilities of cell's invasion, adhesion, and movement. Overall, these differential pathways might contribute to an understanding of the molecular determinants that drive grade progress of astrocytomas.

### Driver genes in the entire process of malignant progression were associated with patient prognosis

**Figure 2:**
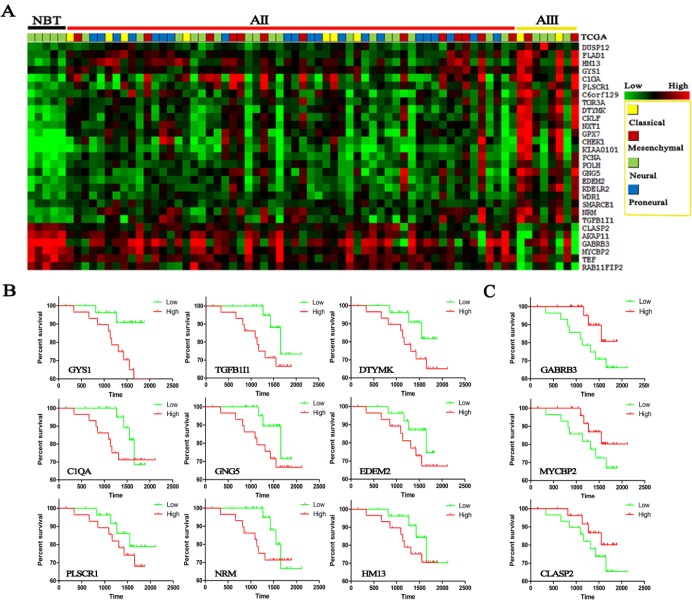
Heat maps were made from candidate genes on 5 NBTs, 58 AIIs and 8 AIIIs in CGGA dataset **(A)** Transcripts levels of 29 candidate genes were identified in three databases and significantly increased with increasing grades (data on the other two databases are supplied in supplementary table 2); TCGA subtypes were given in different colors. Survival analysis was performed by Kaplan-meier plot on candidate genes: **(B)** up-regulated genes; **(C)** down-regulated genes.

To address the molecular foundations of the entire process of grade progression, the differentially expressed genes were overlapped between step1 and step2 in three databases and finally 23 up-regulated and 6 down-regulated candidate genes were identified with a gradual deregulation from NBT to AII, to AIII (*p*<0.05) (Figure-2A, supplementary table 2). We applied the classification system of TCGA to our CGGA samples [10, 14] and found all of neural subtype (5/5 NBTs, 100%) were categorized into NBT. Proneural subtype, not found in NBT and AIII, was the highest percentage of AII (21/58, 36.2%). Mensenchymal and classical subtypes formed an increasing proportion of astrocytomas with increasing grades (0% in NBT, 34.5% in AII and 50% in AIII). The same distribution of subtypes was also found on GSE16011 and REMBRANDT databases (supplementary Figure-1A). This finding indicates that these candidate genes might also contribute to the subtype transition.

Surprisingly, Kaplan–Meier survival analysis found that almost all of these driver genes were associated with patients' prognosis, in spite of not reaching statistical significance in some genes (Figure-2B). However, except for KPS, cox repression analysis showed that there were no significant correlation between patient survival and age, sex, *TP53* mutation and even *IDH1* mutation and 1p19q loss (Supplementary Table 3). The median follow-up period for all 58 IIA patients of CGGA was 49.5 months (range, 5-70.1 months). In spite of not reaching statistical significance in some candidate genes, the prognosis trends were pretty obvious and even startlingly consistent in CGGA and GSE16011 databases (Supplementary Figure-1B) that high expression of up-regulated gene indicates a poor survival whereas high expression of down-regulated gene indicates a favorable survival, respectively.

### Candidate genes were further confirmed by RNA-sequencing and validated by qPCR

**Figure 3:**
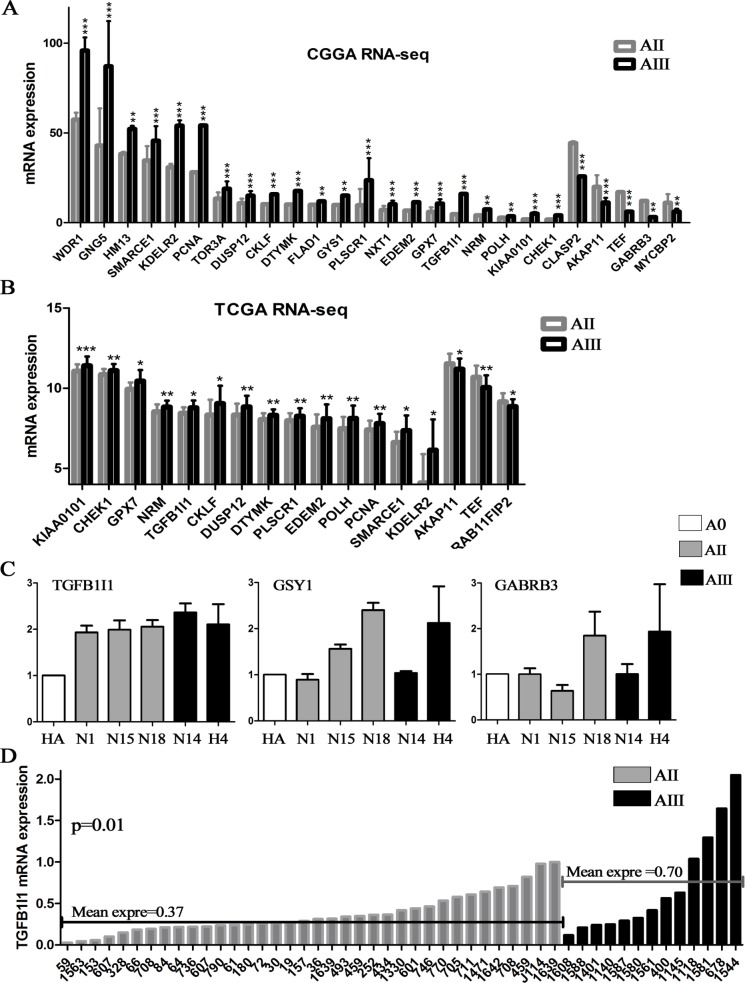
Candidate genes were confirmed on RNA-sequencing data and validated by qPCR **(A and B)** The candidate genes obtained by analysis of microarrays were further confirmed on two RNA-seq database including 57 AII and 71 AIII (CGGA and TCGA); **(C)** The top 3 survival associated genes, GYS1, TGFB1I1 and GABRA3, were validated by qPCR in 6 cell lines (2 commercialized cell lines and 4 primary culture cells). **(D)** The expression level of TGFB1I1 was further validated on additional 49 samples (36 AIIs and 13 AIIIs). *p<0.05, **p<0.01, ***p<0.001

The expression levels of the above candidate genes were projected into 2 independent RNA sequencing databases of glioma (CGGA: 35 AIIs and 13 AIIIs; TCGA: 22 AIIs and 58 AIIIs). 26 of the 29 candidate genes were confirmed in our CGGA database and 17 were further confirmed in TCGA database (Figure-3A and B). Most of these up-regulated genes, *C1QA, CKLF, DTYMK, DUSP12, EDEM2, GPX7, KDELR2, KIAA0101, NRM, PCNA, PLSCR1, POLH, SMARCE1* and *TGFB1I1*, were identified in the malignant progression of a variety of tumors and contributes as an indicator for unfavorable prognosis [15–17].

Finally, we selected the top 3 survival associated genes, *GYS1, TGFB1I1* and *GABRA3*, for validation by qPCR in 6 glioma cell lines (HA and H4, human astrocytes and astrocytoma cell line; N1 and N15, primary culture AII cells; N18, primary culture grade II oligodendroastrocytoma; N14, primary culture grade III oligodendroastrocytoma cell) and additional 49 samples (36 AIIs and 13 AIIIs). Finally, the mRNA expression level of *TGFB1I1* was the best and most consistent with the above results that increasing expression was accompanied by increasing tumor grades (Figure-3C and D). TGFB1I1 was first identified as a TGF-β-inducible gene, and is a member of focal adhesion adaptor proteins. TGF-β-inducible TGFB1I1 has also been shown to function as an oncogene by inducing EMT to promote invasion in cancer cells [17, 18]. More importantly, the up-regulated genes in astrocytomas mostly enriched in EMT associated signaling pathways (actin cytoskeleton, adhesion molecules and TGF-β signaling), suggesting that TGFB1I1-inducible EMT might be involved in the malignant progression of astrocytomas.

### TGFB1I1 was validated on protein level and associated with grade progression and subtype transition

**Figure 4:**
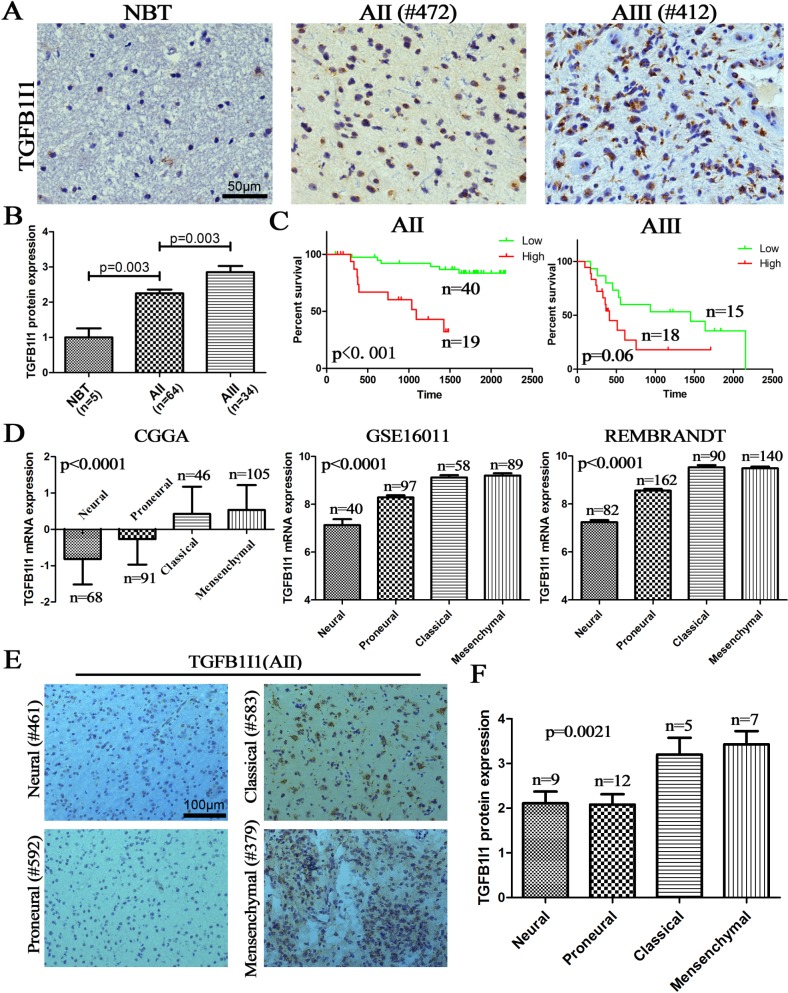
TGFB1I1 protein increased with grade progress and be associated with TCGA subtypes **(A and B)** The protein expression of TGFB1I1 was detected in dependent tissue microarrays by IHC (5 NBTs, 64 AIIs and 34 AIIIs) and the expression level was assessed by semi-quantitative analysis; **(C)** High expression of TGFB1I1 protein indicated a poor survival in AII and AIII. **(D and E)** Either RNA level or protein level, TGFB1I1 was associated with TCGA subtypes and was the highest on mesenchymal subtype.

Analysis of TGFB1I1 expression by IHC in independent tissue microarrays (5 NBTs, 64 AIIs and 34 AIIIs) provided a further level of evidence supporting the key role of TGFB1I1 in malignant progression of astrocytomas. The protein expression of TGFB1I1 was gradually increasing with increasing tumor grades (NBT vs AII at *p*=0.003; AII vs AIII at *p*=0.003) (Figure-4A and B). Importantly, as showed in Figure-4C, we were once again reminded that high expression of TGFB1I1 protein indicated a poor survival in patients with AII and AIII.

Moreover, the transcript level of TGFB1I1 is the highest in mesenchymal subtype in all three databases, suggesting that TGFB1I1 might be associated with increasing invasion and migration (Figure-4D). Indeed, we found that TGFB1I1 was significantly positively correlated with the invasive biomarkers, MMP-2 (*p*=5.8E-39, R^2^=0.46) and MMP-9 (*p*=1.7E-43, R^2^=0.43) (supplementary Figure-2C). A significant positive correlation was observed between TGFB1I1 RNA and protein expression (Figure-4D, E and F). The fact that high expression of TGFB1I1 indicated a poor survival outcome suggests that TGFB1I1 might play important role in malignant progression of astrocytomas by driving cell invasion and migration.

### TGFB1I1 was required for TGF-β stimulated EMT process

**Figure 5:**
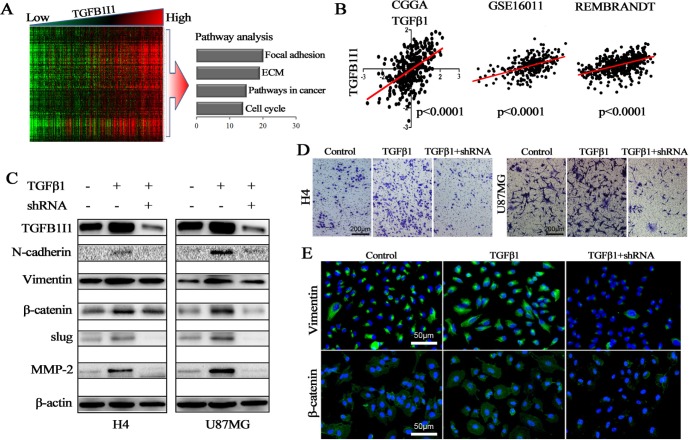
TGFB1I1 regulated TGF-β1 induced EMT **(A)** Correlation analysis performed in 310 samples with mRNA microarrays and a cluster of 308 TGFB1I1 associated genes were obtained (R>0.6, *p*<10^−30^); Pathway analysis was performed using the 308 TGFB1I1 associated genes. **(B)** The expression level of TGFB1I1 was positively correlated with the expression level of TGF-β1 in the three databases. **(C and E)** TGFB1I1 and EMT associated markers were significantly increased in TGF-β1 stimulated cells and TGFB1I1 shRNA resulted in the decreasing expression of N-cadherin, vimentin, slug, β-catenin and MMP-2. **(D)** A significantly decreased invasion was observed in the TGFB1I1 shRNA treated cells compared with TGF-β1 stimulated cells. *p<0.05

TGF-β1 is a classical driver for EMT process [19]. As an important regulator of TGF-β signaling pathway, TGFB1I1 might play important role in TGF-β stimulated EMT process. To confirm the role of TGFB1I1 in EMT process, we performed correlational analyses in 310 samples with genome expression in CGGA database (5 NBTs, 126 grade II, 51 grade III and 128 grade IV gliomas) and obtained a cluster of the top 308 genes (354 probes, supplementary table 5) which were significantly positively correlated with *TGFB1I1* expression (R>0.6, *p*<10^−30^). We then performed KEEG pathway analysis using the 308 *TGFB1I1* associated genes (Figure-5A). We found that gene pathway terms were enriched for cell adhesion and ECM-receptor interaction that was consistent with the above pathway analysis.

**Figure 6:**
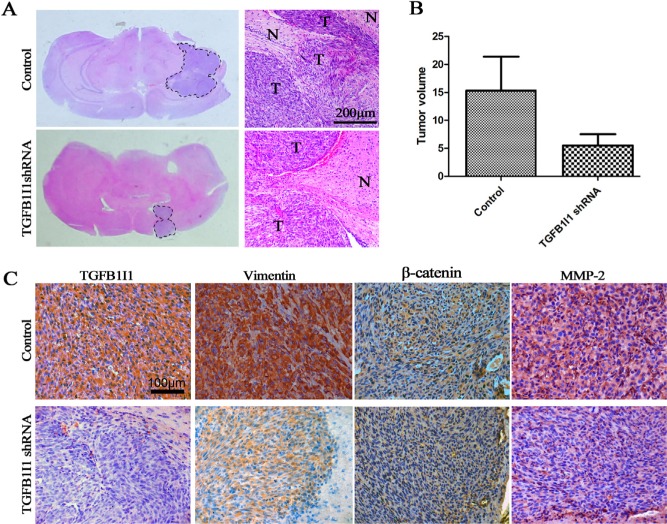
TGFB1I1 promoted tumor progression in vivo **(A and B)** Tumor volume with the TGFB1I1 shRNA was significantly smaller than did control mice. N (control)=6 and N (TGFB1I1shRNA)=4 **(C)** Immunohistochemical staining for the 2 groups of mice, showing decreased vimentin, β-catenin and MMP-2 in mice injected with TGFB1I1 shRNA.

In three databases, we found that there exists a significantly positive correlation between TGFB1I1 and TGF-β1 (Figure-5B). To identify whether or not TGFB1I1 is induced by TGF-β1 in EMT process, we detected TGFB1I1 and EMT markers using western blot and immunofluorescence in H4 and U87 cell lines which were treated with or without TGF-β1 and TGFB1I1 shRNA (Figure-5C and E). The results revealed that TGFB1I1 was significantly increased in TGF-β1 stimulated cells. Similarly, the protein expression of four master markers of EMT, N-cadherin, β-catenin, vimentin, and slug were remarkably elevated in TGF-β1-stimulated cells. The invasive marker, MMP-2, was also increased in TGF-β1-stimulated cells. However, cells treated by TGFB1I1 shRNA displayed a significant reduction in these markers. More importantly, a significantly decreased migration and invasion were observed in the TGFB1I1 shRNA treated cells compared with TGF-β1 stimulated cells (Figure-5D and supplementary Figure-2A). TGF-β1 stimulation also triggered EMT like morphological changes. TGF-β1 treatment led to a pronounced increase of the mesenchymal like cells with elongated size and spindle phenotype (supplementary Figure 2B). TGFB1I1 shRNA transfection significantly counteracted the EMT-like alteration. Next, we examined whether the role of TGFB1I1 also occurs in animal model. U87 control and TGFB1I1 shRNA transfected U87 cells were injected into the brains of a total of 20 nude mice (n=10 per group), and tumor formation was examined after 25 days. Among the 10 mice transplanted with parental U87 cells, 6 mice were found tumors in brains. Whereas, only 4 in the 10 mice transplanted with TGFB1I1 shRNA U87 cells were found tumors. Tumor volume with the TGFB1I1 shRNA was significantly smaller than did control mice (Figure-6B). More importantly, tumors with TGFB1I1 shRNA have a clearer boundary than control tumors with irregular and indefinite border (Figure-6A). Immunohistochemical analysis revealed decreased expression of EMT marker vimentin, β-catenin and MMP-2 in TGFB1I1 shRNA tumors (Figure-6C). These data hit yielded reproducible results that TGFB1I1 play a critical regulator in promoting tumor cell invasion and migration and TGFB1I1 is at least partially responsible for the TGF-β-induced EMT.

## DISCUSSION

Malignant astrocytomas exhibit a relentless malignant progression characterized by widespread invasion throughout the brain. Most patients with low grade gliomas progress to high grade gliomas with increasing malignant degree. However, there also exists malignant progression in equal grade tumors that has been little investigated. The subtypes on the equal grade tumors, like mesenchymal and proneural subtype on GBM, can transit each other [20]. Understanding the mechanism of grade progression and subtypes transition and blocking the main oncogenic pathway are the crux of gliomas therapy. Increasing evidences showed that genetic alterations (mutation, deletion, amplification and overexpression) were involved in the genesis and progression of gliomas [21]. Established biomarkers of astrocytomas, such as *TP53* and *IDH1* mutation, and even recently discovered *TERT* promoter mutations, were considered to be the early event of astrocytomas. In our data, *TP53* and *IDH1* mutation were as high as 50% and 80% in AII whereas only 28% and 39% in AIII. The frequency of *IDH1* and *TP53* mutation in low-grade astrocytoams is similar (or even low) with that in AIII or secondary GBMs suggesting that these alterations might be not associated with the grade progression of astrocytomas (from AII to AIII, sGBM). These two alterations are rather rare in NBT but the highest frequency in AII suggesting that *TP53* and *IDH1* mutations are among early events in astrocytoma development (from NBT to AI or AII). Moreover, patients with *IDH1* mutation frequently possess *TP53* mutation, indicating that this alteration was an earlier event in astrocytomas development than *TP53* mutation. In addition, a small mutation frequency of *PTEN*, 5% reported in other teams, was detected in our samples (3.5% of AII and 17.6% of AIII); the increasing frequency with increasing grades indicates an important role of PTEN signaling in malignant progression of a small subgroup of astrocytomas.

To find the driver genes in malignant progression of astrocytomas, we collected 380 whole genome expression profiles (34 NBTs, 193 IIAs and 153 IIIAs) in three mRNA microarray databases and 2 RNA-sequence databases. Bioinformatics analysis of the RNA expression data followed by pathway analysis revealed that the EMT-associated pathways were most significantly altered along increasing tumor grades with up-regulation of 17 genes. All of the 17 candidate genes were significantly up-regulated with increasing tumor grade and associated with malignant phenotype in various tumors. PCNA and KIAA0101 were classical biomarkers in estimating malignant degree of tumor cells [16, 22]. PCNA, proliferating cell nuclear antigen, is involved in DNA replication and repair in proliferating cells; KIAA0101, a proliferating cell nuclear antigen (PCNA)-associated factor, is involved in the regulation of DNA repair, cell cycle progression, and cell proliferation. DTYMK is increased in the majority of lung adenocarcinomas and elevated DTYMK levels are correlated with poor survival [23]. TGFB1I1 has also been shown to function as an oncogene by inducing EMT to promote invasion in cancer cells as well as in normal breast epithelial cells [24]. Survival analysis showed high expression of these candidate genes indicates poor outcome of patients with astrocytomas. This comprehensive demonstration of these gene changes may serve as a model for studies to understand the complex mechanisms of astrocytomas progression.

Finally, through validation by qPCR and IHC, we focused on TGFB1I1, a critical regulator of EMT process. The activation of EMT program has been proposed as the critical mechanism for the acquisition of malignant phenotypes. Studies in vivo and in vitro have demonstrated that EMT contributes to tumor progression and subtypes transition [25]. Evidences showed that TGF-β signaling is an important inducer of an EMT phenotype in cancer [19]. The central role played by TGF-β in the EMT program is further illustrated by the actions of several TGF-β-inducing transcription factors that facilitate acquisition of a mesenchymal phenotype, such as SMAD, TGFB1I1, RhoA and β-catenin [26]. TGFB1I1, the TGF-β-induced focal adhesion protein, resulted in matrix degradation, cell migration, and invasion via Rac1 regulation of p38 MAPK in TGF-β-treated MCF10A cells [24]. Forced expression of TGFB1I1 is sufficient to induce cytoskeletal organization and led to the formation of ROCK-dependent actin stress fibers [17]. In addition, the promoter of TGFB1I1 contains a CArG element which could be bound by some exogenous factors [27]. TGFB1I1 can shuttle between the nucleus and cytoplasm through an oxidant-sensitive nuclear export sequence by which TGFB1I1 can regulate some gene expression [28]. In our data, the RNA and protein levels of TGFB1I1 were significantly increased along with increasing astrocytoma grades and high expression of TGFB1I1 indicates a poor survival in patients with astrocytomas. Moreover, we found that high expression of TGFB1I1 might contribute to the malignant transition from endothelial-like phenotype to mesenchymal-like phenotype. The RNA and protein levels of TGFB1I1 were the highest in mesenchymal astrocytomas than others subtypes (neural, proneural and classical). By KEEG analysis, TGFB1I1 associated genes were involved in the regulation of cell adhesion and ECM-receptor interactions that were related to the EMT process. These data suggests that TGFB1I1 can promote migration and invasion during TGF-β-induced EMT. As expected, the expression level of TGFβ1 was significantly correlated with TGFB1I1. The invasive biomarkers of gliomas, MMP-2 and MMP-9, have also markedly positive correlation with TGFB1I1. TGF-β1 stimulated induction of N-cadherin, β-catenin, vimentin, slug and even MMP-2 can be blocked by introduction of a specific TGFB1I1 shRNA. The increasing migration and invasion in TGF-β1 stimulated H4 and U87MG cell lines were also blocked by the shRNA plasmid. Finally, these results were further confirmed in vivo experiments.

In conclusion, this finding is new opportunity for understanding the fundamental basis for malignant progression of astrocytomas and the candidate genes might be novel interfering targets for astrocytomas therapy. Our data demonstrates that TGFB1I1 is required for TGF-β stimulated EMT that contributes to malignant progression of astrocytomas. Blocking TGF-β signal pathway by targeting TGFB1I1 might be an effective treatment for astrcytomas.

## METHODS AND MATERIALS

### Samples

Samples were obtained from the Chinese Glioma Genome Atlas (CGGA), including 6 NBTs, 217 AIIs and 40 AIIIs (supplementary table1). The mean age of diagnosis is 38.7 years ranging from 13 to 74 years in all levels of astrocytomas. Patients with AIII have a slightly later median age of onset of about 42.9 years. Males are slightly more commonly affected, with a male to female ratio of about 1.54:1. These samples were used to perform mRNA expression profiles, RNA-sequencing, detection of the established biomarkers, immunohistochemistry and survival analysis. The numbers of sample in each analysis were mentioned in following sections. Normal adult brain samples were obtained after informed consent from patients with severe traumatic brain injury who needed posttrauma surgery and from patients who had undergone surgery for primary epilepsy. All of the patients underwent surgical resection from January 2005 through December 2012. Patients were eligible for the study if their diagnosis was established histologically by 2 neuropathologists according to the 2007 WHO classification guidelines. Tumor tissue samples were obtained by surgical resection before patients underwent radiation and/or chemotherapy. Only samples with 80% tumor cells were selected for analysis. This study was approved by the institutional review boards of all hospitals involved in the study, and written informed consent was obtained from all patients.

### Cell culture and reagents

The human astrocytoma cell lines H4, U87 and human astrocytes (HA) were obtained from the Institute of Biochemistry and Cell Biology, Chinese Academy of Science. Freshly resected tumor tissues (N1 and N15, primary culture AII cells; N18, primary culture grade II oligodendroastrocytoma; N14, primary culture grade III oligodendroastrocytoma cell) were enzymatically and mechanically dissociated into single cells and grown in DMEM/F12 media supplemented with B27 (Invitrogen), EGF (20 ng/ml), and bFGF (20 ng/ml). Cells were maintained in DMEM containing 10% FBS, 50 U/ml penicillin G, and 250 μg/ml streptomycin in a humidified atmosphere containing 5% CO^2^ at 37°C. For TGFβ1 stimulation, cells were treated with 6 ng/ml TGFβ1 (Peprotech) for 24 h before assay.

### shRNA transfection

H4 and U87MG cells were transfected with TGFB1I1 shRNA (a pool of 3 target-specific lentiviral vector plasmids, Santa Crusz, USA, sc-37685-SH) and control vector (sc-108060) with lipofectamine (Invitrogen) according to the manufacturer's instructions. Stably transfected cells were selected for with 0.5 μg/ml (H4) and 0.4 μg/ml (U87) puromycin (Invitrogen) for 2 weeks, after which clones were selected and amplified.

### Detection on the established biomarkers

Genomic DNA was isolated from frozen tumor tissues by using the QIAamp DNA Mini Kit (Qiagen). The genomic region spanning wild-type R132 of *IDH1* (182 AIIs and 33 AIIIs) and MGMT promoter methylation (23 AIIs and 24 AIIIs) were analyzed by pyrosequencing (QIAGEN, Germany). *PTEN* (140 AIIs and 17 AIIIs) and *TP53* mutation (149 AIIs and 18 AIIIs) was identified by exon direct sequencing and sanger sequencing, respectively. EGFR amplificantion and 1p19q loss (6 NBTs, 217 AIIs, and 40 AIIIs) was identified by fluorescence in situ hybridization (FISH).

### RNA expression data

Our whole-genome mRNA expression microarray data (5 NBTs, 58 AIIs, 8 AIIIs) and RNA-sequencing data (35 AIIs and 13 AIIIs) were deposited in the CGGA database. The other two whole-genome mRNA expression microarray data were downloaded from the repository for molecular brain neoplasia data (REMBRANDT, http://caintegrator-info.nci.nih.gov/rembrandt: 21 NBTs, 65 AIIs and 58 AIIIs) and GSE16011 data (http://www.ncbi.nlm.nih.gov/geo/query/acc.cgi?acc=GSE 16011: 8 NBTs, 13 IIAs and 16 IIIAs). TCGA (The Cancer Genome Atlas) RNA-sequencing data was downloaded from TCGA database (http://cancergenome.nih.gov: 22 AIIs and 58 AIIIs).

### qPCR

The expression levels of TGFB1I1, GSY1 and GABRB3 in 6 cell lines and 49 samples were analyzed with real-time quantitative PCR using the SYBR Supermix Kit (Bio-Rad, Hercules, CA). PCR included the following components: 100 nM each primer, diluted cDNA templates, and iQ SYBR Green supermix. PCR efficiency was examined by serially diluting the template cDNA, and the melting curve data were collected to check PCR specificity. Each cDNA sample was run as triplicates. The GAPDH primer was included in every plate to avoid sample variations. The relative mRNA level was presented as unit values of 2^ [Ct (GAPDH) – Ct (gene of interest)]. The primer sequences for TGFB1I1, GSY1 and GABRB3 are listed in Supplementary Table 4.

### Immunohistochemistry and immunocytofluorescent

Immunohistochemistry (IHC) of paraffin sections in tissue microarrays and immunocytofluorescent (IF) in H4 cell line were performed as previously described [29]. Briefly, the sections were incubated with primary antibody, TGFB1I1 (Santa Crusz, 1:200 dilution), beta-catenin and vimentin (Cell Signaling Technology, 1:200 dilution), MMP-2 (ABCAM at a concentration of 5 μg/ml) overnight at 4°C, then incubated with a biotinylated secondary antibody (1:200 dilution) at room temperature for 1 h, followed by the incubation with ABC-peroxidase reagent (1:200 dilution, Vector, USA) for an additional 1 h. After washing with Tris-buffer, the sections were stained with DAB for 5 min, rinsed in water and counterstained with hematoxylin. IF was performed on H4 cell line. Briefly, cells were incubated with primary antibody for 1 h at room temperature. FITC-labeled secondary antibodies were added at 1:100 dilution, and the cells were then incubated for another 30 min. Nuclei were stained with 4,6-diamidino-2-phenylindole (DAPI; Invitrogen).

### Western blot

Whole-cell lysates were prepared using RIPA buffer. Equal amounts of total protein (30 μg) from cell lysates were loaded on a 10% SDS/PAGE gel, transferred to a PVDF membrane (Millipore), and detected using an ECL Western Blotting Detection System (Biorad). Antibodies were primary antibodies TGFB1I1 (Santa Crusz, 1:500) N-cadherin, beta-catenin, slug, vimentin (Cell Signaling Technology, 1:1000 dilution) and MMP-2 (ABCAM, at a concentration of 5 μg/ml). β-actin (sigma, 1:5000 dilution) was used as the loading control. Secondary antibodies used were goat anti-rabbit IgG-HRP and goat anti-mouse IgG-HRP (Zhongshan Gold Bridge Biotechnology).

### Invasion assay

Transwell invasion assays were performed using transwell filters (Costar, USA) coated with Matrigel (3.9 μg/μl, 60-80 μl) on the upper surface of the polycarbonic membrane (diameter 6.5 mm, pore size 8 μm). A total of 1×10^5^ cells were plated onto the upper chamber of the transwell plates with 2% FBS in DMEM. To the lower chambers of the wells was added 10% FBS in DMEM. Following 24 h incubation, the non-invading cells were removed from the upper surfaces of the invasion membranes and the cells on the lower surface were stained with crystal violet. The average number of cells per field was determined by counting cells in 6 random fields per well.

### Wound assay

Cells were grown in 6-well plates with complete medium. After 90% confluence was reached, the wound was created by a germ-free 100 μl pipette tip in the monolayer. The cells were washed with PBS and grown in 2% FBS media for 24 h. The wounds were observed under a phase contrast microscope (IX81, Olympus). The images were analysed by drawing lines at the wound edges. The width of the scratch was measured at 0, 8, 18 and 24 h post-treatment.

### Vivo experiment

BALB/c female athymic mice were implanted in the brain with U87 wild-type and shRNA transfected cells. Briefly, mice were anesthetized with 5% chloral hydrate and cells were implanted using cranial guide screws as previously described [30]. A TJ-4A Syringe Pump Controller and microinfusion syringe pump (1 μl/min) were used to implant 3 × 10^6^ cells into the brain of mice. Mice were sacrificed after 25 days simultaneously. Brains were extracted and fixed in 10% formalin for 24 hours, embedded in paraffin, and sectioned into 5 μm slices

### Statistical analysis

SPSS version 13.0 was used for all statistical analyses. Differentially expression genes were detected by unpaired Student's t-test. The χ^2^ test was applied for statistical analysis of the correlation for 2 independent variables. Kaplan-Meier survival analysis was used to estimate the survival distributions. The log-rank test was applied to assess the statistical significance between stratified survival groups using the GraphPad Prism version 4.0 statistical software. KEGG pathway and GO analysis was performed using DAVID (http://david.abcc.ncifcrf.gov/). Heat maps of different grade astrocytomas were constructed by Gene Cluster 3.0 and Gene Tree View software. A two-sided *p* value <0.05 was regarded as significant.

## SUPPLEMENTARY FIGURES AND TABLES




