# Supplementary material for: Multidimensional analysis of gene expression reveals TGFB1I1-induced EMT contributes to malignant progression of astrocytomas
Source: Oncotarget. 2014 Dec 31;5(24):12593–606. doi: 10.18632/oncotarget.2518 (PMC4350345; doi:10.18632/oncotarget.2518)
Supplement: Supplementary file 1 [file oncotarget-05-12593-s001.pdf]

SUPPLEMENTARY FIGURES AND TABLES

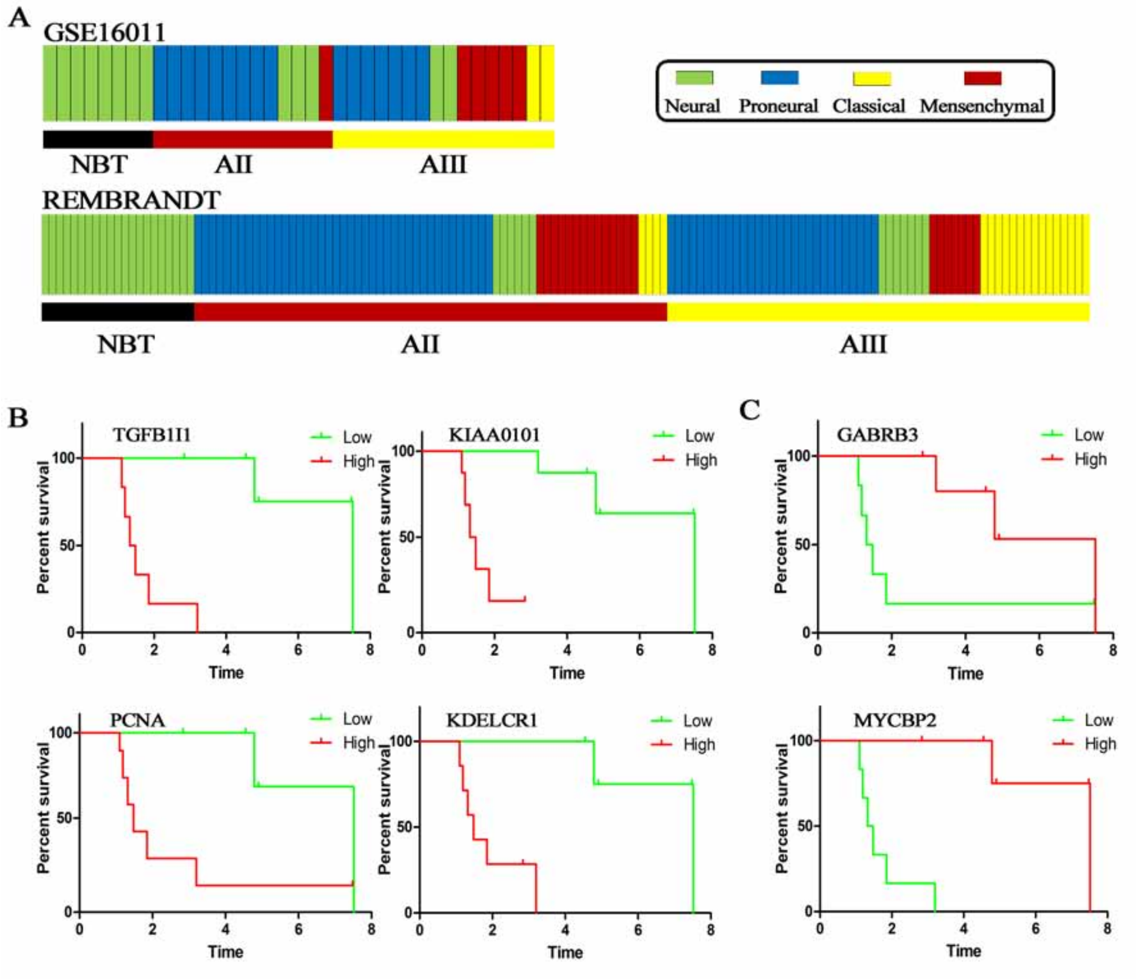

Supplementary Figure S1: (A) The classification system of the TCGA was projected into samples of GSE16011 and REMBRANDT. (B and C) Survival analysis was performed by Kaplan-meier plot on candidate genes in GSE16011 database: (B) up-regulated genes; (C) down-regulated genes.

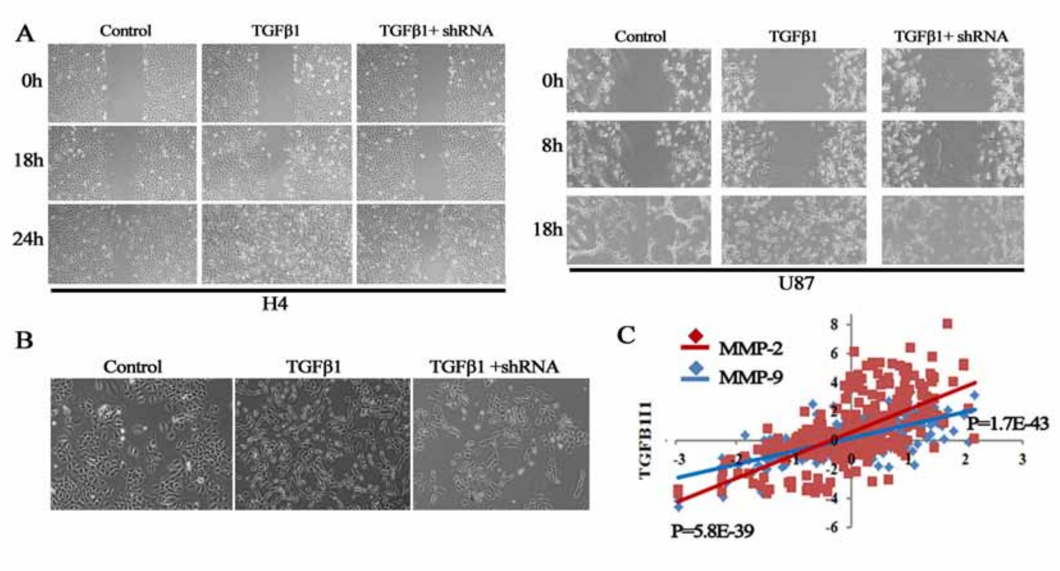

Supplementary Figure S2: (A) Wound healing assay demonstrated that TGF-β1 treatment accelerated wound close of H4 and U87 cells. TGFB1I1 knockdown reversed the TGF-β1 enhanced migration. (B) H4 cells with longer branches were more scattered upon TGF-β1 treatment, whereas these phenotypes were reversed by TGFB1I1 specific shRNA. (C) TGFB1I1 expression level was positively correlated with the invasion markers, MMP-2/9.

Supplementary Table S1. Clinical characteristics on samples of the study in CGGA database

Variables	NBT(n=6)	AII(n=217)	AIII(n=40)	Total*(n=257)
Mean age(range)	50.8(29–69)	37.9(13–72)	42.9(18–74)	38.7(13–74)
Gender(%)				
male	4(66.7%)	133(61.3%)	23(57.5%)	156(60.7%)
female	2(33.3%)	84(38.7%)	17(42.5%)	101(39.3%)

*patients with gliomas

Supplementary Table S3. Cox regression analysis of information of 58 AII

Variable	Number of case	P value
**Age**		.082
<37	29(50%)	
≥37	29(50%)	
**Sex**		.373
Male	37(64%)	
Female	21(36%)	
**KPS**		.021
≤80	15(26%)	
>80	43(74%)	
**1p**		.064
No loss	49(89%)	
Loss	6(11%)	
**19q**		.056
No loss	48(87%)	
Loss	7(13%)	
**P53**		.410
Mutation	19(46%)	
Wild type	22(54%)	
**IDH1**		.400
Mutation	42(74%)	
Wild type	15(26%)	

Supplementary Table S4. The primer sequences for TGFB1I1, GSY1 and GABRB3

**TGFB1I1**		**Length**	**TM**
Forward Primer	5′-TACAGCACGGTATGCAAGCC-3	20	62.6
Reverse Primer	5′-GCAACCGATCTAGCTCACAGAG-3	22	62.4
**GYS1**			
Forward Primer	5′-GCGCTCACGTCTTCACTACTG-3	21	63
Reverse Primer	5′-TCCAGATGCCCATAAAAATGGC-3	22	61
**GABRB3**			
Forward Primer	5′-GATAAAAGGCTCGCCTATTCTGG-3	23	60.8
Reverse Primer	5′-GATCATGCGGTTTTTCACTGTC-3	22	60.1

Supplementary Table S5.

Probe	symbol	R	*P* value
A_23_P96568	FLNA	0.781335	5.06E-65
A_23_P76006	SERPINH1	0.777496	5.32E-64
A_23_P323563	PLEKHG2	0.761359	6.43E-60
A_23_P337934	FBLIM1	0.757977	4.19E-59
A_23_P65240	COL4A1	0.755438	1.68E-58
A_23_P205031	COL4A2	0.754683	2.53E-58
A_32_P28284	TPM4	0.753448	4.93E-58
A_23_P254888	ZYX	0.748616	6.44E-57
A_23_P33277	BMP1	0.745535	3.22E-56
A_32_P21993	TPM4	0.745166	3.89E-56
A_23_P8055	NRM	0.744228	6.32E-56
A_32_P56001	CD93	0.743519	9.10E-56
A_24_P129417	BMP1	0.742898	1.25E-55
A_23_P144896	PDLIM7	0.739159	8.35E-55
A_23_P162970	IPO4	0.738226	1.33E-54
A_24_P935491	COL3A1	0.737289	2.13E-54
A_23_P142533	COL3A1	0.736034	3.98E-54
A_23_P165722	EIF4E2	0.735531	5.10E-54
A_23_P5273	SBNO2	0.734928	6.87E-54
A_23_P331479	KIAA1949	0.732909	1.85E-53
A_23_P210811	CD93	0.731868	3.08E-53
A_23_P141974	TPM4	0.73138	3.91E-53
A_23_P201628	LAMC1	0.729192	1.12E-52
A_23_P207520	COL1A1	0.724157	1.23E-51
A_23_P52697	CD248	0.721753	3.80E-51
A_23_P251695	NXT1	0.716641	3.99E-50
A_23_P63038	LEPRE1	0.716604	4.06E-50
A_23_P49338	TNFRSF12A	0.714997	8.41E-50
A_23_P79622	FKBP7	0.714104	1.26E-49
A_24_P402242	COL3A1	0.712531	2.55E-49
A_23_P432360	PMM2	0.712457	2.64E-49
A_23_P23191	HSPG2	0.712181	2.98E-49
A_23_P92261	ECE2	0.712058	3.15E-49
A_24_P68342	COL4A1	0.711743	3.62E-49
A_23_P111995	LOXL2	0.711066	4.90E-49
A_24_P39508	CMTM3	0.709098	1.17E-48

(*Continued*)

Probe	symbol	R	*P* value
A_24_P42517	KDELR2	0.708837	1.31E-48
A_23_P113212	TMEM45A	0.707834	2.04E-48
A_24_P211565	C1QTNF6	0.70388	1.14E-47
A_24_P106145	WDR1	0.70291	1.73E-47
A_23_P303210	IKBIP	0.701946	2.61E-47
A_23_P393598	CD276	0.700762	4.33E-47
A_24_P234196	RRM2	0.70043	4.98E-47
A_23_P22224	EIF4EBP1	0.69646	2.65E-46
A_24_P382187	IGFBP4	0.695064	4.75E-46
A_23_P256956	KIF20A	0.694206	6.78E-46
A_23_P200443	SHC1	0.694195	6.81E-46
A_23_P205789	GABPB1	0.69195	1.72E-45
A_23_P395438	HTRA3	0.688736	6.37E-45
A_23_P151506	PLEK2	0.687723	9.59E-45
A_24_P739344	NOX4	0.687011	1.28E-44
A_23_P50349	TRIP10	0.686572	1.52E-44
A_23_P83818	COL5A1	0.686418	1.62E-44
A_23_P136978	SRPX2	0.685945	1.96E-44
A_23_P27096	PFN1	0.685644	2.21E-44
A_23_P10542	HTRA3	0.685505	2.34E-44
A_23_P95470	CD151	0.68546	2.38E-44
A_24_P914479	SNX5	0.685292	2.54E-44
A_24_P59220	POTEF	0.685046	2.81E-44
A_24_P277934	COL1A2	0.684512	3.47E-44
A_23_P165848	EMILIN1	0.683691	4.81E-44
A_23_P54313	CD276	0.683477	5.24E-44
A_23_P107421	TK1	0.683222	5.80E-44
A_23_P211233	COL6A2	0.682608	7.39E-44
A_23_P19938	KDELR2	0.682567	7.51E-44
A_23_P207896	CSNK1D	0.682025	9.30E-44
A_24_P13533	PPIL5	0.680739	1.54E-43
A_23_P163787	MMP2	0.68043	1.74E-43
A_32_P96719	SHCBP1	0.680295	1.84E-43
A_24_P246173	MYO9B	0.679072	2.96E-43
A_23_P115872	CEP55	0.67855	3.63E-43
A_32_P234604	PFN1	0.678392	3.86E-43
A_24_P162718	LMNA	0.678345	3.93E-43

(*Continued*)

Probe	symbol	R	*P* value
A_24_P135322	NRP1	0.677862	4.74E-43
A_23_P24104	PLAU	0.677204	6.11E-43
A_23_P74138	TAGLN2	0.677157	6.22E-43
A_23_P254271	TUBB6	0.676966	6.70E-43
A_23_P88865	CMTM3	0.676955	6.73E-43
A_23_P105028	ATL3	0.676853	7.00E-43
A_23_P34835	LMNA	0.676815	7.10E-43
A_23_P168788	PLOD3	0.676469	8.12E-43
A_23_P30884	CLIC1	0.676228	8.91E-43
A_23_P253434	FNDC3B	0.674565	1.69E-42
A_23_P12514	RHOC	0.67408	2.03E-42
A_24_P297539	UBE2C	0.673926	2.15E-42
A_23_P131866	AURKA	0.673517	2.52E-42
A_23_P16944	SDC1	0.672413	3.83E-42
A_32_P194848	TAGLN2	0.671747	4.93E-42
A_23_P127926	WEE1	0.671521	5.37E-42
A_24_P188071	TUBA1C	0.671435	5.55E-42
A_23_P88331	DLGAP5	0.671431	5.56E-42
A_23_P406424	RHOC	0.671417	5.58E-42
A_24_P57426	COL18A1	0.671154	6.17E-42
A_23_P34126	BGN	0.670763	7.15E-42
A_24_P186414	ZFAND3	0.670729	7.24E-42
A_24_P399888	CENPM	0.669638	1.09E-41
A_23_P83328	ENG	0.669158	1.31E-41
A_23_P92132	IFRD2	0.668983	1.40E-41
A_23_P434919	RAB42	0.668767	1.51E-41
A_24_P322741	IL10RB	0.668721	1.54E-41
A_23_P212458	SEC61A1	0.668334	1.78E-41
A_23_P118815	BIRC5	0.667256	2.66E-41
A_23_P62115	TIMP1	0.666212	3.92E-41
A_24_P257099	HJURP	0.665546	5.01E-41
A_23_P62831	FAM176B	0.665474	5.15E-41
A_23_P67708	TCF3	0.6653	5.49E-41
A_23_P57588	GTSE1	0.663984	8.91E-41
A_23_P145357	BAK1	0.66318	1.20E-40
A_23_P49597	PLSCR3	0.663165	1.20E-40
A_23_P310956	COL6A2	0.662477	1.55E-40

(*Continued*)

Probe	symbol	R	*P* value
A_23_P154675	SNRPB	0.662273	1.67E-40
A_23_P204702	TMBIM6	0.661743	2.02E-40
A_24_P370156	MAN2B1	0.660629	3.03E-40
A_23_P40347	HM13	0.660564	3.10E-40
A_23_P57417	MMP11	0.660027	3.77E-40
A_23_P47148	NOX4	0.659978	3.83E-40
A_23_P137103	EIF4A1	0.659548	4.48E-40
A_23_P109452	CHEK2	0.659316	4.87E-40
A_24_P60930	BMP1	0.659286	4.93E-40
A_23_P201483	MAPKAPK2	0.659014	5.43E-40
A_23_P51853	ZDHHC18	0.658911	5.64E-40
A_23_P149992	PDLIM1	0.658871	5.72E-40
A_23_P11874	MPZL1	0.658134	7.45E-40
A_23_P251499	PCOLCE	0.657124	1.07E-39
A_23_P98898	CDK2	0.656645	1.27E-39
A_23_P94795	TEAD4	0.655766	1.74E-39
A_23_P124417	BUB1	0.654966	2.31E-39
A_23_P250122	FAM20C	0.653978	3.28E-39
A_23_P214185	UBE2J1	0.653742	3.56E-39
A_23_P5392	TP53I3	0.653536	3.83E-39
A_23_P94422	MELK	0.653432	3.97E-39
A_23_P72651	ECSCR	0.653097	4.47E-39
A_23_P65757	CCNB2	0.652382	5.75E-39
A_32_P149432	EIF4A1	0.652342	5.83E-39
A_23_P40174	MMP9	0.652335	5.85E-39
A_23_P385861	CDCA2	0.652204	6.12E-39
A_23_P78664	DDX39	0.652039	6.49E-39
A_32_P66843	C19orf69	0.651816	7.01E-39
A_23_P201047	THBS3	0.651368	8.20E-39
A_32_P167239	AFAP1L1	0.650891	9.69E-39
A_23_P200507	CNIH4	0.650772	1.01E-38
A_23_P44836	NT5DC2	0.650724	1.03E-38
A_23_P23457	FBLIM1	0.650484	1.12E-38
A_23_P156327	TGFBI	0.65025	1.21E-38
A_23_P137532	PLOD1	0.650231	1.22E-38
A_23_P101796	SYDE1	0.649856	1.39E-38
A_23_P211212	COL18A1	0.649454	1.60E-38

(*Continued*)

Probe	symbol	R	*P* value
A_23_P140907	TMEM8A	0.648987	1.88E-38
A_24_P318656	ITGB3	0.648464	2.26E-38
A_23_P57927	HYAL2	0.648156	2.51E-38
A_23_P49627	MRC2	0.648086	2.57E-38
A_23_P161194	VIM	0.648056	2.60E-38
A_24_P119745	FN1	0.647336	3.33E-38
A_23_P52738	DCPS	0.647107	3.60E-38
A_24_P12413	TRAM2	0.646241	4.85E-38
A_23_P256334	ITGA1	0.6458	5.65E-38
A_24_P242391	MPZL1	0.645369	6.54E-38
A_23_P146644	ANXA2	0.645	7.42E-38
A_23_P57379	CDC45L	0.644891	7.70E-38
A_32_P94798	ANXA2	0.644438	8.99E-38
A_24_P413884	CENPA	0.643415	1.27E-37
A_23_P73982	TMEM48	0.643399	1.28E-37
A_23_P60856	TSPAN4	0.642982	1.48E-37
A_23_P122815	CALU	0.642544	1.71E-37
A_23_P158593	COL5A1	0.642377	1.81E-37
A_24_P96403	RUNX1	0.642235	1.90E-37
A_23_P210763	JAG1	0.64218	1.94E-37
A_23_P117852	KIAA0101	0.641936	2.10E-37
A_24_P270144	CD63	0.641544	2.40E-37
A_23_P100486	VKORC1	0.641536	2.41E-37
A_24_P85539	FN1	0.640669	3.22E-37
A_23_P93321	TCF19	0.640146	3.84E-37
A_23_P50477	BCL2L12	0.639782	4.34E-37
A_23_P258281	GPR172A	0.639384	4.96E-37
A_23_P311616	JMJD6	0.638923	5.78E-37
A_23_P255016	UGGT1	0.638776	6.08E-37
A_23_P141965	HAUS8	0.638638	6.36E-37
A_23_P78802	PRKD2	0.638514	6.63E-37
A_23_P42575	CALD1	0.638354	6.99E-37
A_24_P204244	ANXA2P1	0.638207	7.34E-37
A_23_P122052	GPX8	0.637626	8.91E-37
A_23_P4913	KDELR1	0.637223	1.02E-36
A_23_P107412	P4HB	0.637051	1.08E-36
A_23_P340848	PTGIR	0.636938	1.12E-36

(*Continued*)

Probe	symbol	R	*P* value
A_23_P153745	IFI30	0.636765	1.19E-36
A_23_P132793	MANF	0.636673	1.22E-36
A_23_P308581	C6orf153	0.636353	1.36E-36
A_23_P216501	TPM2	0.635572	1.76E-36
A_23_P138507	CDC2	0.635369	1.88E-36
A_23_P106532	CHST14	0.634568	2.45E-36
A_23_P49878	FAM64A	0.634376	2.61E-36
A_23_P76515	LASS5	0.634231	2.74E-36
A_23_P54834	NIP7	0.633987	2.96E-36
A_23_P64232	ZNF259	0.633734	3.22E-36
A_24_P245646	TP53RK	0.633592	3.37E-36
A_23_P74097	TCEB3	0.633483	3.50E-36
A_23_P124084	LOXL1	0.633236	3.79E-36
A_23_P87513	ATP6V0A2	0.632418	4.95E-36
A_23_P138796	AMOTL1	0.632336	5.09E-36
A_23_P67360	PLEKHA4	0.632202	5.31E-36
A_24_P6921	LOC541471	0.631604	6.46E-36
A_24_P408047	PLEKHA4	0.631526	6.62E-36
A_24_P198598	PML	0.631117	7.56E-36
A_32_P192842	LOC100293208	0.630598	8.95E-36
A_24_P273143	NCRNA00152	0.630441	9.42E-36
A_23_P155815	NCAPG	0.630426	9.46E-36
A_23_P50108	NDC80	0.630289	9.89E-36
A_24_P322354	SKA1	0.630286	9.90E-36
A_23_P212844	TACC3	0.630282	9.91E-36
A_23_P212696	FSTL1	0.629751	1.18E-35
A_24_P932887	SPOCD1	0.62966	1.21E-35
A_24_P56130	MYL6	0.629324	1.35E-35
A_23_P4919	KDELR1	0.629241	1.39E-35
A_32_P32254	COL6A1	0.629073	1.46E-35
A_23_P73972	GPX7	0.628896	1.55E-35
A_23_P41246	MFSD10	0.628584	1.71E-35
A_23_P48099	NUP37	0.628502	1.76E-35
A_23_P119943	IGFBP2	0.628344	1.85E-35
A_23_P161190	VIM	0.628291	1.88E-35
A_23_P205449	CDCA4	0.628165	1.96E-35
A_32_P108254	FAM20A	0.62804	2.04E-35

(*Continued*)

Probe	symbol	R	*P* value
A_23_P148475	KIF4A	0.628002	2.07E-35
A_23_P103672	NES	0.627453	2.46E-35
A_32_P525524	ITPRIPL1	0.627383	2.52E-35
A_24_P313504	PLK1	0.62738	2.52E-35
A_24_P304723	PPIB	0.626746	3.09E-35
A_23_P203882	MMP19	0.626672	3.16E-35
A_23_P153889	PGLS	0.626642	3.19E-35
A_23_P60079	ANGPT2	0.62644	3.41E-35
A_23_P344973	MYL6	0.626283	3.58E-35
A_23_P208293	PVRL2	0.62573	4.27E-35
A_24_P407866	SC65	0.625005	5.38E-35
A_23_P143190	MYBL2	0.624778	5.78E-35
A_24_P82106	MMP14	0.624373	6.58E-35
A_24_P876522	GPX8	0.623448	8.81E-35
A_23_P88731	RAD51	0.623263	9.34E-35
A_23_P16523	GDF15	0.623072	9.92E-35
A_23_P316601	RIT1	0.622465	1.20E-34
A_23_P423864	PHC2	0.622431	1.21E-34
A_23_P9614	NDUFA4L2	0.622314	1.26E-34
A_23_P370625	SEPN1	0.622086	1.35E-34
A_23_P67288	CALR	0.621819	1.47E-34
A_23_P102769	C20orf72	0.62149	1.63E-34
A_23_P58321	CCNA2	0.621157	1.81E-34
A_23_P165360	ASB1	0.621129	1.83E-34
A_23_P411814	OSTC	0.620901	1.96E-34
A_23_P50456	POLD1	0.620652	2.12E-34
A_23_P368896	SNX12	0.619799	2.77E-34
A_32_P54442	CHPF2	0.619615	2.93E-34
A_24_P352952	FAM20A	0.619137	3.40E-34
A_23_P130182	AURKB	0.61885	3.72E-34
A_23_P154500	DNMT3A	0.618648	3.96E-34
A_24_P223124	FNDC3B	0.618248	4.48E-34
A_23_P350467	MAPKAPK2	0.618245	4.48E-34
A_24_P932435	ANGPT2	0.618115	4.67E-34
A_23_P129614	NUDT21	0.617884	5.01E-34
A_23_P36562	ITGA5	0.617842	5.08E-34
A_23_P126266	HLX	0.617795	5.15E-34

(*Continued*)

Probe	symbol	R	*P* value
A_23_P20566	TPM2	0.617247	6.10E-34
A_23_P4662	BCL3	0.617166	6.26E-34
A_23_P209183	GLT25D1	0.616844	6.91E-34
A_32_P210202	E2F7	0.616778	7.05E-34
A_23_P136805	ARHGAP11A	0.61658	7.50E-34
A_23_P25224	CSDA	0.616093	8.71E-34
A_24_P320545	PTK7	0.615815	9.49E-34
A_23_P45475	GLA	0.615814	9.49E-34
A_23_P100127	CASC5	0.615745	9.69E-34
A_23_P16469	PLAUR	0.615719	9.77E-34
A_23_P334608	GUSB	0.615161	1.16E-33
A_23_P133656	LAMA4	0.614568	1.39E-33
A_24_P918907	LOC154761	0.61426	1.53E-33
A_23_P132277	MCM5	0.614233	1.54E-33
A_23_P32707	ESPL1	0.614162	1.57E-33
A_23_P408328	TRIOBP	0.614004	1.65E-33
A_23_P67913	GMPPA	0.613705	1.81E-33
A_23_P114232	PRDX4	0.613663	1.83E-33
A_23_P163458	EHD4	0.613418	1.97E-33
A_24_P146892	ORAI1	0.613243	2.08E-33
A_23_P81805	VEGFA	0.61311	2.17E-33
A_24_P37903	LOX	0.613028	2.22E-33
A_23_P431388	SPOCD1	0.61297	2.26E-33
A_23_P333605	ENPEP	0.612943	2.28E-33
A_23_P163087	NID2	0.612795	2.39E-33
A_32_P827528	S1PR2	0.612466	2.64E-33
A_23_P133956	KIFC1	0.612388	2.70E-33
A_23_P202327	ADAM12	0.612246	2.82E-33
A_23_P68610	TPX2	0.612128	2.92E-33
A_23_P375	CDCA8	0.612124	2.93E-33
A_24_P29260	MGAT4B	0.612092	2.95E-33
A_32_P135902	EIF4A1	0.612067	2.98E-33
A_23_P118174	PLK1	0.611996	3.04E-33
A_24_P372862	ETV6	0.611978	3.06E-33
A_24_P410686	KDELR2	0.611706	3.32E-33
A_23_P107206	STAT3	0.61142	3.62E-33
A_23_P305100	KIAA1919	0.610176	5.27E-33

(*Continued*)

Probe	symbol	R	*P* value
A_23_P94030	LAMB1	0.610134	5.34E-33
A_23_P387057	TUBB	0.609956	5.63E-33
A_23_P16673	CNN2	0.609561	6.34E-33
A_23_P47304	CASP5	0.609391	6.67E-33
A_23_P25626	C13orf34	0.609357	6.74E-33
A_23_P118061	CKLF	0.609274	6.91E-33
A_23_P487	UCK2	0.609208	7.05E-33
A_24_P67988	FRMD8	0.609192	7.08E-33
A_23_P382688	FAM122B	0.608969	7.57E-33
A_23_P366394	ZAK	0.608866	7.81E-33
A_23_P257144	PXDN	0.608712	8.18E-33
A_24_P390793	EIF4A1	0.608685	8.24E-33
A_23_P34788	KIF2C	0.608539	8.61E-33
A_23_P130900	NCLN	0.608384	9.02E-33
A_32_P49188	ALG9	0.608029	1.00E-32
A_23_P111888	CTHRC1	0.608028	1.00E-32
A_32_P62997	PBK	0.607801	1.07E-32
A_23_P7636	PTTG1	0.607678	1.11E-32
A_23_P153197	TGIF1	0.607544	1.16E-32
A_23_P103628	HEATR1	0.607366	1.22E-32
A_23_P500799	CASP6	0.60717	1.30E-32
A_24_P323434	CDCA2	0.606826	1.44E-32
A_24_P417984	PPP1R14B	0.606824	1.44E-32
A_23_P28733	RBL1	0.606633	1.52E-32
A_23_P18579	PTTG2	0.606311	1.67E-32
A_23_P56328	PLVAP	0.606238	1.71E-32
A_24_P14156	NDC80	0.605825	1.93E-32
A_24_P323598	ESCO2	0.605737	1.98E-32
A_23_P145584	UBE2H	0.605639	2.04E-32
A_23_P200928	NID1	0.60556	2.09E-32
A_23_P120458	RNF114	0.605156	2.36E-32
A_23_P57497	MYH9	0.605137	2.37E-32
A_23_P416656	MYO1C	0.604932	2.52E-32
A_24_P19337	ASXL1	0.604725	2.68E-32
A_23_P145863	S100A11	0.604679	2.71E-32
A_23_P306655	AMIGO3	0.604676	2.72E-32
A_23_P99063	LUM	0.604009	3.31E-32

(*Continued*)

Probe	symbol	R	*P* value
A_24_P921366	CALD1	0.603998	3.32E-32
A_23_P93677	C7orf42	0.603994	3.32E-32
A_23_P316741	TSPAN4	0.603805	3.51E-32
A_23_P404259	GPX8	0.603185	4.21E-32
A_23_P69188	DPH3	0.602883	4.60E-32
A_23_P149200	CDC20	0.602592	5.01E-32
A_23_P57736	CMTM6	0.60252	5.12E-32
A_24_P334130	FN1	0.602375	5.34E-32
A_23_P387943	CASP2	0.602077	5.83E-32
A_23_P164451	TBX2	0.601902	6.13E-32
A_23_P122197	CCNB1	0.601871	6.19E-32
A_23_P22957	SH3GLB1	0.601548	6.80E-32
A_24_P399680	C20orf108	0.601533	6.83E-32
A_23_P162540	KCTD10	0.601413	7.08E-32
A_23_P124733	COQ2	0.60141	7.08E-32
A_23_P201778	PTPN7	0.600693	8.73E-32
A_23_P253752	FAM54A	0.600548	9.10E-32
A_24_P462899	C6orf173	0.600523	9.17E-32
A_23_P52676	CATSPER1	0.600382	9.55E-32
A_23_P113994	KIRREL	0.600349	9.65E-32
A_24_P416079	NUSAP1	0.600112	1.03E-31
A_23_P25121	FKBP11	0.600076	1.04E-31
